# Comparative Natural History of Visual Function From Patients With Biallelic Variants in *BBS1* and *BBS10*

**DOI:** 10.1167/iovs.62.15.26

**Published:** 2021-12-23

**Authors:** Monika K. Grudzinska Pechhacker, Samuel G. Jacobson, Arlene V. Drack, Matteo Di Scipio, Ine Strubbe, Wanda Pfeifer, Jacque L. Duncan, Helene Dollfus, Nathalie Goetz, Jean Muller, Andrea L. Vincent, Tomas S. Aleman, Anupreet Tumber, Caroline Van Cauwenbergh, Elfride De Baere, Emma Bedoukian, Bart P. Leroy, Jason T. Maynes, Francis L. Munier, Erika Tavares, Eman Saleh, Ajoy Vincent, Elise Heon

**Affiliations:** 1 Department of Ophthalmology and Vision Sciences, The Hospital for Sick Children, Toronto, Canada; 2 Department of Ophthalmology and Vision Sciences, University of Toronto, Toronto, Canada; 3 Department of Ophthalmology, Scheie Eye Institute, Perelman School of Medicine, University of Pennsylvania, Philadelphia, Pennsylvania, United States; 4 Department of Ophthalmology, Institute for Vision Research, University of Iowa, Iowa City, Iowa, United States; 5 Genetics and Genome Biology, The Hospital for Sick Children, Toronto, Canada; 6 Department of Ophthalmology, Ghent University Hospital & Department of Head and Skin, Ghent University, Ghent, Belgium; 7 Department of Ophthalmology, University of California, San Francisco, San Francisco, California, United States; 8 CARGO (*Centre de référence pour les affections rares génétiques*), IGMA *Institut de Génétqiue Médicale d'Alsace*, Hôpitaux Universitaires de Strasbourg, Strasbourg, France; 9 UMRS_1112, IGMA (*Institut de génétique Médicale d'Alsace*) Université de Strasbourg, Strasbourg, France; 10 Laboratoire de diagnostique génétique, IGMA (*Institut de génétique Médicale d'Alsace*) Hôpitaux Universitaires de Strasbourg, Strasbourg, France; 11 Department of Ophthalmology, New Zealand National Eye Centre, University of Auckland, Auckland, New Zealand; 12 Eye Department, Greenlane Clinical Centre, Auckland District Health Board, Auckland, New Zealand; 13 Center for Advanced Retinal and Ocular Therapeutics, Perelman School of Medicine, Philadelphia, Pennsylvania, United States; 14 Scheie Eye Institute at the Perelman Center for Advanced Medicine, Philadelphia, Pennsylvania, United States; 15 Division of Ophthalmology, The Children's Hospital of Philadelphia, Philadelphia, Pennsylvania, United States; 16 Center for Medical Genetics, Ghent University and Ghent University Hospital, Ghent, Belgium; 17 Center for Molecular Therapeutics, Children's Hospital of Philadelphia, Philadelphia, Pennsylvania, United States; 18 Department of Anesthesia and Pain Medicine, The Hospital for Sick Children, Toronto, Ontario, Canada; 19 Departments of Biochemistry and Anesthesiology and Pain Medicine, University of Toronto, Program in Molecular Medicine, The Hospital for Sick Children, Toronto, Canada; 20 Jules-Gonin Eye Hospital, Fondation Asile des Aveugles, University of Lausanne, Lausanne, Switzerland

**Keywords:** blindness, Bardet Biedl syndrome, retinal degeneration, genetics, natural history, end points

## Abstract

**Purpose:**

The purpose of this study was to compare the natural history of visual function change in cohorts of patients affected with retinal degeneration due to biallelic variants in Bardet-Biedl syndrome genes: *BBS1* and *BBS10*.

**Methods:**

Patients were recruited from nine academic centers from six countries (Belgium, Canada, France, New Zealand, Switzerland, and the United States). Inclusion criteria were: (1) female or male patients with a clinical diagnosis of retinal dystrophy, (2) biallelic disease-causing variants in *BBS1* or *BBS10*, and (3) measures of visual function for at least one visit. Retrospective data collected included genotypes, age, onset of symptoms, and best corrected visual acuity (VA). When possible, data on refractive error, fundus images and autofluorescence (FAF), optical coherence tomography (OCT), Goldmann kinetic perimetry (VF), electroretinography (ERG), and the systemic phenotype were collected.

**Results:**

Sixty-seven individuals had variants in *BBS1* (*n* = 38; 20 female patients and 18 male patients); or *BBS10* (*n* = 29; 14 female patients and 15 male patients). Missense variants were the most common type of variants for patients with *BBS1*, whereas frameshift variants were most common for *BBS10.* When ERGs were recordable, rod-cone dystrophy (RCD) was observed in 82% (23/28) of patients with *BBS1* and 73% (8/11) of patients with *BBS10*; cone-rod dystrophy (CORD) was seen in 18% of patients with *BBS1* only, and cone dystrophy (COD) was only seen in 3 patients with *BBS10* (27%). ERGs were nondetectable earlier in patients with *BBS10* than in patients with *BBS1*. Similarly, VA and VF declined more rapidly in patients with *BBS10* compared to patients with *BBS1*.

**Conclusions:**

Retinal degeneration appears earlier and is more severe in *BBS10* cases as compared to those with *BBS1* variants. The course of change of visual function appears to relate to genetic subtypes of BBS.

Bardet‐Biedl syndrome (BBS) has a broad range of clinical features that typically include severe photoreceptor degeneration often combined with truncal obesity, postaxial polydactyly, autism‐like behavior, cognitive impairment, hypogonadism, and renal anomalies, among other features.^1^^–^^6^ Biallelic variants have been identified in 24 *BBS* genes,^7^^,^^8^ where *BBS1* and *BBS10* together are the most commonly involved.^9^^,^^10^ BBS is considered a ciliopathy as the underlying genes are expressed in primary cilia.^11^^,^^12^ BBS1, together with seven other BBS proteins form a protein complex named BBSome, a key regulator of the ciliary membrane proteome, important for ciliary transport.^13^^,^^14^ Whereas, BBS10 protein form a complex with two other chaperonin-like proteins responsible for BBSome assembly.^15^

Bardet‐Biedl syndrome is phenotypically and genetically heterogeneous, and demonstrates considerable phenotypic and mechanistic overlap with other ciliopathies, such as Joubert syndrome and Senior-Løken syndrome.^1^^,^^2^^,^^4^^,^^5^^,^^16^^–^^25^ The systemic features of BBS vary among affected individuals but photoreceptor dysfunction is a constant finding. In some patients, retinal degeneration may be the only manifestation of BBS-related variants (e.g. *BBS1* and *C8orf37*),^26^^,^^27^ which was also observed for other ciliopathy genes (e.g. *CEP290*) causing syndromic and non-syndromic retinal degeneration.^28^ Hence, genetic testing is required to identify the pathogenic variants in *BBS* gene to confirm *BBS*-related disease.

The retinal degeneration associated with BBS is usually early and severe. The phenotypes observed include rod‐cone dystrophy (RCD), cone-rod dystrophy (CORD), or cone dystrophy (COD).^29^^–^^34^ Central and peripheral visual function loss are most noticeable by the second or third decade of life when 73% of affected individuals become legally blind.^1^^,^^30^ Studies of murine models suggest that the photoreceptor degeneration could be due to the accumulation of non-outer segment proteins in the outer segment, rather than failure of protein delivery to the outer segment.^35^

It is important to identify *BBS*-related disease especially that a recent mice model study suggests that *BBS10*-related disease could be treatable, (Drack AV, et al. IOVS 2021; 62: ARVO E-Abstract 1178). Lessons learned from early gene therapy studies for Leber congenital amaurosis (LCA) due to biallelic pathogenic variants in *RPE65* gene highlight the importance of natural history information in patient selection and choosing the useful outcome measures to best interpret results of clinical treatment trials.^36^^,^^37^

This international collaborative effort allowed collection of data from patients with retinal degeneration and biallelic variants in the two most commonly involved BBS genes; *BBS1* and *BBS10* (hereon referred to as patients with *BBS1* and patients with *BBS10*). We compared the ocular phenotype of patients with *BBS1* and patients with *BBS10* to gain insight into the natural history of visual function loss over time.

## Methods

This retrospective study involved nine participating centers across six countries (Belgium, Canada, France, New Zealand, Switzerland, and the United States,) and was approved by the institutional ethics review board of each participating center and the procedures followed the tenets of the Declaration of Helsinki. Patients were identified through the respective internal databases. Inclusion criteria were: (1) female or male subjects with retinal degeneration, (2) biallelic pathogenic or presumed pathogenic variants in *BBS1* or *BBS10* genes, and (3) availability of ocular and systemic phenotype information for at least one visit. The patient's evaluation and genetic counseling were provided according to the best standard of care practice of each institution. For those countries in the European Union, the Oviedo Convention and the Treaty of Lisbon were honored. Data collected and analyzed included de-identified demographic information, ocular and medical history, systemic phenotype, DNA genetic results and parameters of the ocular phenotype (visual acuity [VA], Goldmann kinetic perimetry [VF], and electroretinography [ERG]). In selected cases, optical coherence tomography (OCT) scans were also reviewed. Full data sets were not available for all subjects for all testing parameters and some patients only had data from one visit (Table 1).

**Table 1.** Demographic Information and Summary of Ocular Assessments

Parameter/Study Group	BBS1 (*n* = 38)	BBS10-RCD* (*n* = 26)	BBS10*-COD* (*n* = 3)
**Sex**, *n* (%)			
Female	20 (51)	13 (50)	1 (33)
Male	18 (49)	13 (50)	2 (67)
**Ethnicity**, *n* (%)			
Caucasian	28 (76)	18 (62)	3 (100)
Asian	2 (5)	0 (0)	0 (0)
Hispanic	1 (3)		
Unknown	6 (16)	10 (38)	0 (0)
**Age at initial visit**, years			
Mean	18.2	12.9	19.5
Range	(3.0-49.0)	(2.0-45.0)	(10.2-33.4)
**Age at last visit**, years			
Mean	27.3	21.3	32
Range	(5.0-58.0)	(2.0-53.0)	(25.0-41.1)
**Observation time**			
Mean, years	10.1	8.3	12
Range, years	(0-33.9)	(0-19.0)	(7.7-15.2)
No. of visits			
Mean, *n*	5	6	5
Range, *n*	(1.0-15.0)	(1.0-13.0)	(3-8)
**Refraction**, *n* (%)			
Myopia	13 (50)	13 (56)	3 (100%)
Hyperopia	11 (42)	8 (30)	0 (0)
Emmetropia	2 (8)	3 (13)	0 (0)
Astigmatism >2 D	12 (46)	13 (48)	1 (33)
**Cataract**, *n* (%)	11 (31)	9 (50)	1 (50)
Age of onset	27.2	18.4	33.4
Mean, years	8.5-47.8	13.6-25.0	NA
Range, years		13 (81)	NA
**Nyctalopia**, *n* (%)			
Age of onset	24 (86)	13 (81)	0 (0) at age 33.4
Mean, years	19.4	10	NA
Range, years	5.0-35.9	2.0-19.3	NA
**Photophobia**, *n* (%)			
Age of onset	18 (72)	13 (81)	1 (33)
Mean, years	22.5	15.2	36.0
Range, years	8.0-47.8	12.1-26.5	NA

COD, cone dystrophy; RCD, rod-cone dystrophy; *n*, number *includes individuals with non-detectable ERG at first visit but symptomatology of RCD; NA, not applicable.

Data availability: BBS1 (*n* = 26 for refraction, *n* = 35 for cataract, *n* = 28 for nyctalopia, and *n* = 25 for photophobia); BBS10-COD (*n* = 2 for cataract); severe BBS10 (*n* = 23 for refraction, *n* = 19 for cataract, *n* = 16 for nyctalopia, and *n* = 17 for photophobia).

### Visual Function Assessment

Data available from full field ERGs were collected from patient’ charts at different ages when possible. ERGs were performed incorporating standards of The International Society for Clinical Electrophysiology of Vision (ISCEV) using Diagnosys LLC system (Canada, France, and the United States) or RETI-port system, Roland Consult (Belgium and New Zealand).^38^^,^^39^ ERG results were interpreted by each principal investigator and categorized as RCD, CORD, COD, or non-detectable (ND) at first visit. The RCD pattern referred to the reduced rod and cone photoreceptor responses with predominantly reduced rod ERGs; the CORD pattern referred to reduction in cone and rod ERG responses but predominantly reduced cone responses; and the COD phenotype referred to a reduction in cone responses with preserved rod responses. ND referred to severely reduced responses not discernable from noise.

### Visual Acuity

The methods of VA assessment included preferential looking Teller acuity cards for preverbal children and Snellen acuity charts, or decimal (France), for older individuals. VA data for children ≤ 5 years of age were excluded due to poor reliability. All VA measurements were converted to logarithm of the minimum angle of resolution (LogMAR). Because refractive errors were not documented for all patients in some cases it was uncertain if the best correction was used. For this reason, we refer to VA and not BCVA. For the purpose of data analyses, patients who could only count fingers (CFs), perceive hand motion (HM), had only light perception (LP) or no light perception (NLP) were assigned LogMAR values of 2.6, 2.7, 2.8, and 2.9, respectively.^40^^,^^41^ Acuity of each eye was measured separately but, for analyses, VA results are presented as an average of both eyes.

For patients with ≥ 5 data points, simple linear regression analyses of VA by age were performed for data from both *BBS1* and *BBS10* cohorts.

### Refraction

Refractions were available for 62% of patients with *BBS1* and 93% of patients with *BBS10*. Myopia was defined as a spherical equivalent < −0.5 diopters (D), hyperopia as a spherical equivalent ≥ +1.0 D and significant astigmatism as ≥ 2 D of cylinder. Results are presented as a mean spherical equivalent from both eyes.

### Visual Fields

Goldmann kinetic perimetry assessments were collected at different ages for each eye. The outcome measure was the diameter across horizontal meridians for each stimulus tested (III4e and V4e). Due to symmetry, the end point was the average value from both eyes. Longitudinal and cross-section analyses of the VF to the III4e and V4e stimuli were available for some individuals and simple linear regression analyses were performed on both cohorts as described for VA. A point plot graph also was performed on both cohorts with III4e data, as described below.

### Data Analysis

Data were collected and summarized using descriptive measures, including means with standard deviations (SDs) and medians with ranges for continuous variables such as age, and frequencies and percentages for categorical variables, such as gender. Simple linear regression analyses were analyzed in base R and graphed in ggplot2 (https://ggplot2.tidyverse.org) in RStudio (http://www.rstudio.com/).^42^^,^^43^

### Genetic Analysis

Genetic diagnosis was performed through different laboratories: Molecular Genetics Laboratory of Ghent University Hospital (Belgium); the Diagnostic Genetics Laboratory at Strasbourg University Hospital (France); John & Marcia Carver Nonprofit Genetic Testing Laboratory and Prevention Genetics (United States); and the Genetic Diagnostics in Tubingen and other CLIA-approved laboratories (for Canada, New Zealand, and the United States). Cases for which some data were previously published are referenced in Table 2. Mutations were verified to adhere to the latest nomenclature of the Human Genome Variation Society recommendations (*BBS1* [NM_024649] and *BBS10* [NM_024685.4], https://varnomen.hgvs.org).^44^ Prediction of the pathogenicity of variants used tools publicly available. Our approach to assess the pathogenicity of variants using predictive algorithms is outlined in Table 3. The structure of BBS10 protein was modeled with Phyre2, using the cryo-EM structure of the mammalian chaperonin TRiC/CCT (PDB ID 3IYG) and the X-ray crystal structures of GroEL/GroES (PDB IDs 1SVT and 1Q3S) as templates.^45^^–^^49^

**Table 2.** Summary of Systemic Features and Variants in all Cases

**Pt. no.**	Digit Anomaly	Kidney Anomalies	Liver Anomalies	Hearing	Cardiac Abnormalities	Diabetes	Ataxia/Poor Coordinatio	Cognitive Impairment	Dev Delay/Learning Disabilities	Speech Disorder/Delay	Behavioral Abnormalities	Genital Abnormalities	Variant 1	Variant 2	Reference
***BBS1***															
**1**	+	NA	NA	−	−	−	−	NA	NA	−	−	−	Arg160Gln	Arg160Gln	^1^
**2**	NA	+	+	−	−	−	−	−	+	+	−	−	Met390Arg	Asn524del	^1^ ^,^ ^2^
**3**	+	−	+	−	−	+	−	NA	+	−	−	−	Met390Arg	Met390Arg	^1^
**4**	+	+	+	+	−	−	+	NA	+	+	+	NA	Ile330Thr	Arg483*	^1^ ^–^ ^3^
**5**	+	−	+	−	−	+	+	−	+	+	NA	NA	Met1?	Met1?	^1^
**6**	+	−	+	−	−	+	+	−	+	+	NA	+	Met1?	Met1?	^1^
**7**	+	−	−	−	−	−	+	−	+	+	NA	NA	Met390Arg	Met390Arg	^1^
**8**	+	−	−	−	−	−	−	+	+	+	NA	+	c. 724-8_726del, p?	Met390Arg	^1^ ^,^ ^3^ ^,^ ^4^
**9**	+	+	−	−	−	−	+	−	+	−	NA	+	Met390Arg	Met390Arg	^1^
**10**	+	+	+	−	−	+	+	−	+	+	NA	NA	Ile296Thrfs*7	Ile296Thrfs*7	^1^ ^,^ ^4^
**11**	+	NA	NA	−	+	−	−	NA	−	−	NA	+	Met390Arg	Met390Arg	^1^
**12**	+	−	−	−	−	NA	−	NA	NA	−	+	NA	Met390Arg	Leu505Profs*52	^1^ ^,^ ^3^
**13**	+	−	−	−	−	−	+	−	+	−	NA	NA	Met390Arg	Leu505Profs*52	^1^ ^,^ ^3^
**14**	+	NA	−	−	−	−	+	+	+	−	NA	NA	Met390Arg	c. 1473+2T>C, p?	^1^
**15**	+	+	−	−	−	−	−	+	+	+	NA	NA	Met390Arg	Met390Arg	^1^
**16**	+	−	−	−	−	−	−	+	+	−	NA	NA	Met390Arg	Met390Arg	^1^
**17**	NA	+	+	NA	NA	+	NA	NA	NA	NA	NA	NA	Met390Arg	c.1340-1G>T, p?	New
**18**	+	−	−	+	+	+	−	−	−	+	−	+	Met390Arg	Met390Arg	
**19**	+	−	−	−	+	−	−	−	−	−	−	NA	Met390Arg	Met390Arg	
**20**	NA	−	−	+	−	−	NA	+	+	−	NA	NA	Met390Arg	Met390Arg	
**21**	−	−	−	−	−	−	NA	−	−	−	+	NA	Met390Arg	Met390Arg	
**22**	+	+	−	+	−	−	NA	+	+	−	NA	−	Met390Arg	Met390Arg	
**23**	NA	−	NA	NA	NA	NA	NA	NA	NA	NA	NA	NA	Met390Arg	Met390Arg	
**24**	+	−	−	+	−	−	−	−	−	−	−	−	Met390Arg	Met390Arg	
**25**	−	−	−	+	−	−	−	−	−	−	−	−	Met390Arg	Met390Arg	
**26**	+	+	NA	−	−	+	−	+	+	+	−	−	c. 1340-1G>T, p?	c. 1473+2T>C, p?	New
**27**	+	−	−	−	−	−	−	−	−	−	+	+	Met390Arg	Met390Arg	
**28**	+	+	−	+	−	−	+	+	+	−	+	−	Glu384*	Met390Arg	New
**29**	+	+	−	+	+	−	−	+	−	−	+	−	Met390Arg	Met390Arg	
**59**	+	NA	NA	NA	NA	NA	NA	NA	NA	NA	NA	NA	Met390Arg	Met390Arg	
**60**	+	NA	NA	NA	NA	NA	NA	NA	NA	NA	NA	NA	Met390Arg	Met390Arg	
**61**	+	−	−	−	−	−	−	−	+	−	−	−	Met390Arg	Met390Arg	
**62**	+	+	−	−	−	−	−	−	−	−	+	−	Met390Arg	Met390Arg	
**63**	+	+	−	−	−	−	−	+	+	−	−	−	Met390Arg	Met390Arg	
**64**	+	+	−	−	−	−	−	+	+	−	+	−	Met390Arg	Met390Arg	
**65**	+	+	−	−	−	+	−	+	+	−	−	+	Ile296Thrfs*7	Ile296Thrfs*7	
**66**	+	+	−	−	−	−	−	+	+	−	−	−	Met390Arg	Met390Arg	
**67**	+	−	+	−	−	−	+	+	+	−	−	−	Met390Arg	Thr405Thrfs*46	^5^

**Table 2.** Continued

**Pt. no.**	Digit Anomaly	Kidney Anomalies	Liver Anomalies	Hearing	Cardiac Abnormalities	Diabetes	Ataxia/Poor Coordinatio	Cognitive Impairment	Dev Delay/Learning Disabilities	Speech Disorder/Delay	Behavioral Abnormalities	Genital Abnormalities	Variant 1	Variant 2	Reference
***BBS10***															
**30**	+	−	−	+	−	+	+	+	+	+	+	+	Arg49Trp	Arg49Trp	^1^ ^,^ ^4^
**31**	+	+	+	−	−	−	−	−	+	−	−	NA	Cys91Trp	Val707*	^1^ ^,^ ^2^ ^,^ ^4^
**32**	+	+	+	−	−	−	+	+	+	+	+	+	Cys91Leufs*5	Glu104Lysfs*7	^1^ ^,^ ^3^ ^,^ ^4^
**33**	+	+	−	−	−	−	NA	+	+	+	+	+	Cys91Trp	Ala474Metfs*10	^1^ ^–^ ^4^
**34**	+	+	−	−	−	−	NA	−	+	+	−	−	Cys91Trp	Ala474Metfs*10	^1^ ^–^ ^4^
**35**	+	+	−	−	−	+	+	+	+	+	NA	+	Cys91Leufs*5	Tyr559*	^1^ ^,^ ^4^
**36**	+	−	NA	−	NA	NA	NA	+	+	NA	+	+	Cys91Leufs*5	Tyr469*	^1^ ^,^ ^3^
**37**	+	+	−	−	−	−	+	−	+	+	−	−	Cys91Leufs*5	Tyr469*	^1^ ^,^ ^3^
**38**	+	+	−	−	−	−	+	+	+	+	−	+	Cys91Leufs*5	Tyr469*	^1^ ^,^ ^3^
**39**	+	−	−	−	−	−	−	−	−	−	−	NA	Ile407Thr	Ile407Thr	
**40**	−	−	−	−	−	−	−	−	NA	NA	NA	−	Glu61*	Leu76Phe	
**41**	−	−	−	−	−	−	−	+	NA	NA	NA	−	Glu61*	Leu76Phe	
**42**	+	+	−	−	−	+	−	NA	NA	NA	NA	+	Cys91Leufs*5	Tyr177*	
**43**	−	−	+	−	−	+	−	+	NA	NA	+	NA	Ser303Argfs*3	His656Leufs*4	^4^
**44**	+	+	−	−	−	−	+	+	+	−	+	−	Cys91Leufs*5	Cys91Leufs*5	^3^
45^(^*^)^	NA	NA	NA	NA	NA	NA	NA	NA	NA	NA	NA	NA	Asn364Thrfs*5	Thr524Alafs*13	
**46^(^**†**^)^**	+	NA	NA	NA	NA	NA	NA	NA	NA	NA	NA	NA	Tyr589*	Tyr589*	
**47**	NA	NA	NA	NA	NA	NA	NA	NA	NA	NA	NA	NA	Leu76Ilefs*34	Cys91Leufs*5	
**48**	NA	NA	NA	NA	NA	NA	NA	NA	NA	NA	NA	NA	Cys91Leufs*5	Phe86Leu	
**49**	NA	NA	NA	NA	NA	NA	NA	NA	NA	NA	NA	NA	Arg49Trp	Cys91Leufs*5	
**50**	+	NA	NA	NA	NA	NA	NA	NA	+	NA	NA	NA	Ser444Valfs*44	Arg49Trp	
**51**	NA	NA	NA	NA	NA	NA	NA	NA	NA	NA	NA	NA	Cys91Leufs*5	Cys91Leufs*5	
**52**	+	−	−	+	−	−	NA	+	+	−	NA	+	Arg49Trp	Arg49Trp	
**53**	+	−	−	+	−	−	NA	+	+	−	NA	NA	Arg49Trp	Arg49Trp	
**54**	+	+	−	+	+	−	NA	+	+	−	NA	−	Arg49Trp	Arg49Trp	
**55**	+	+	NA	+	−	−	−	−	−	−	−	−	Leu414Ser	Cys91Leufs*5	^4^
**56**	+	−	−	−	−	−	+	−	−	−	−	−	Cys91Leufs*5	Pro350Ilefs*11	
**57**	+	−	−	−	−	−	−	+	−	−	+	+	Arg49Trp	Arg49Trp	
**58**	+	+	+	−	+	+	−	−	−	−	−	+	Cys91Leufs*5	Cys91Leufs*5	^4^
^(^*^)^ Hirschsprung disease; ^(^†^)^ heterotaxia. NA: not available. –: not present, +: present.															
**Summary of systemic phenotypes in patients with *BBS1* and patients with *BBS10***															
				
Feature				*BBS1*, *n* (%)				*BBS10*, *n* (%)			
				
Digit anomalies				32/34 (94)				21/24 (88)			
Developmental delay/learning disabilities				23/32 (72)				14/19 (74)			
Cognitive impairment				14/29 (48)				13/21 (62)			
Kidney anomalies				15/33 (45)				12/22 (55)			
Behavioral abnormalities				8/21 (38)				7/15 (47)			
Ataxia/poor coordination				10/31 (32)				7/16 (44)			
Genital anomalies				7/22 (32)				10/18 (56)			
Speech disorder				10/34 (29)				7/17 (41)			
Liver anomalies				8/32 (25)				4/20 (20)			
Hearing problems				8/34 (24)				5/22 (23)			
Diabetes				8/34 (24)				5/21 (24)			
Cardiac anomalies				4/34 (12)				2/21 (10)			

NA, not applicable.

**Table 3.** Reported Variants in BBS1 and BBS10 and Their Corresponding Predictive Scores

Gene	Variant	Predicted Effect	dbSNP^*^	PhyloP^†^	SIFT/PolyPhen-2^3‡^	Allele frequency (GnomAD)^4§^	Splicing^‖^	Access Number^6¶^ (ClinVar or Uniprot)
BBS1	c.1A>G, p.(Met1?)	Startloss	rs1306821707	NA	NA	0.000003989	Truncation from start	Lik. Pat.-RCV000671318.1
	c.479G>A, p.(Arg160Gln)	Missense, splicing	rs376894444	5.13	0.07/0.947	0.0000278	NNSPLICE: 91.5%	Pat. RCV001074216.1
	c.480-1G>C, p.?	Likely skip exon 6	rs1057516933	NA	NA	0	NNSPLICE: -100.0%	Lik. Pat.-RCV000409654.1
	c.724-8_726del, p.?	Likely skip exon 9	NA	NA	NA	0	Premature stop	Lik. Pat. RCV001073554.1
	c.887del, p.(Ile296Thrfs*7)	Frameshift stop	rs794727006	NA	NA	0	Premature stop	Pat. VCV000193740.2
	c.989T>C, p.(Ile330Thr)	Missense	NA	4.48	0.3/0.730	0	NA	Lik. Pat-VAR_066278
	c.1150G>T, p.(Glu384*)	Nonsense	NA	4.56	NA	0	Premature stop	Novel
	c.1169T>G, p.(Met390Arg)	Missense	rs113624356	3.76	0.01/ 0.347	0.001570	NA	Pat. RCV000787785.1
	c.1214_1215insSVA, p.(Thr405Thrfs*46)	Frameshift stop	NA	NA	NA	NA	NA	Pat. SCV001245066.1
	c.1340-1G>T, p.?	Likely skip exon 14	NA	NA	NA	0	NNSPLICE: -100.0%	Lik. Pat. RCV000669016.1
	c.1447C>T, p.(Arg483*)	Nonsense	NA	4.48	NA	NA	Premature stop	Pat. RCV000804705.1
	c.1473+2T>C, p.?	Likely skip exon 14	NA	4.0	NA	0	NNSPLICE: -100.0%	Lik.Pat. VCV000866282.1
	c.1514_1515del, p.(Leu505Profs*52)	Frameshift stop	rs775769424	NA	NA	0.00001395	Premature stop	Pat. RCV000410181.2
	c.1568_1570del, p.(Asn524del)	Inframe deletion	rs863224782	4.24	NA	0.000003976	NA	Unc. RCV000198771.1
BBS10	c.145C>T, p.(Arg49Trp)	Missense	rs768933093	1.17	0/0.998	0.00009558	NA	Pat. RCV000799037.2
	c.181G>T, p.(Glu61*)	Nonsense	NA	1.09	NA	0	Premature stop	Novel
	c. 224_225dup, p.(Leu76Ilefs*34)	Inframe deletion	NA	NA	NA	0	Premature stop	Novel
	c.226C>T, p.(Leu76Phe)	Missense	rs767638924	2.1	0.16/0.905	0.000004177	NA	Novel
	c.258T>A, p.(Phe86Leu)	Missense	NA	0.12	0/0.962	0	NA	Novel
	c.271dup, p.(Cys91Leufs*5)	Nonsense	rs549625604	NA	NA	0.0005642	Premature stop	Pat. RCV001074512.1
	c.273C>G, p.(Cys91Trp)	Missense	rs148374859	0.45	0/0.928	0.00002816	NA	Pat. RCV000023803.5
	c.310_311del, p.(Glu104Lysfs*7)	Frameshift stop	NA	NA	NA	0	Premature stop	Novel
	c.531C>A, p.(Tyr177*)	Nonsense	rs863224522	0.29	NA	0	Premature stop	Lik. Pat. RCV000409505.1
	c.909_912del, p.(Ser303Argfs*3)	Frameshift stop	rs780059308	NA	NA	0.00001774	Premature stop	Pat. RCV000811417.1
	c.1044_1045del, p.(Pro350Ilefs*11)	Frameshift stop	rs587777837	NA	NA	0	Premature stop	Pat. RCV000023802.7
	c.1091del, p.(Asn364Thrfs*5)	Frameshift stop	rs727503818	NA	NA	0.00006738	Premature stop	Pat RCV001004383.1
	c.1220T>C, p.(Ile407Thr)	Missense	rs750164736	0.85	0.19/0.006	0.000007958	NA	Novel
	c.1241T>C, p.(Leu414Ser)	Missense	rs786204575	2.06	0.3/0.339	0.000003979	NA	Lik. Pat. RCV000169317.1
	c.1330del, p.(Ser444Valfs*44)	Frameshift stop	NA	NA	NA	0	Premature stop	Novel
	c.1407T>G, p.(Tyr469*)	Nonsense	rs1356713858	0.61	NA	0.000006977	Premature stop	Pat. RCV000779832.1

**Table 3.** Continued

Gene	Variant	Predicted Effect	dbSNP^*^	PhyloP^†^	SIFT/PolyPhen-2^3‡^	Allele frequency (GnomAD)^4§^	Splicing^‖^	Access Number^6¶^ (ClinVar or Uniprot)
	c.1420_1432del, p.(Ala474Metfs*10)	Frameshift stop	NA	NA	NA	NA	Premature stop	Novel
	c.1566_1569dup, p.(Thr524Alafs*13)	Frameshift stop	NA	NA	NA	0	Premature stop	Novel
	c.1677C>A, p.(Tyr559*)	Nonsense	rs375413604	NA	NA	0.00004252	Premature stop	Pat. RCV000477827.2
	c.1767C>A, p.(Tyr589*)	Nonsense	NA	NA	NA	NA	Premature stop	Lik. Pat. RCV000760514.1
	c.1967del, p.(His656Leufs*4)	Frameshift stop	NA	NA	NA	0	Premature stop	Novel
	c.2119_2120del, p.(Val707*)	Nonsense	rs775950661	NA	NA	0.00006015	Premature stop	Pat. RCV000665753.1

* Database of single nucleotide polymorphisms (dbSNP; http://www.ncbi.nlm.nih.gov/SNP/).

† PhyloP basewise conservation score derived from alignment of 46 vertebrate species (range = -14.1 to 6.4). Higher levels are more conserved (Pollard KS, Hubisz MJ, Siepel A. *Detection of non-neutral substitution rates on mammalian phylogenies* Genome Res. 2010 Jan;20(1):110-21. PMID: 19858363).

‡ Missense predictors: SIFT(predicts whether an **amino acid substitution affects protein function** based on sequence homology and the physical properties of amino acids; https://sift.bii.a-star.edu.sg/)/Polyphen(predicts possible impact of an amino acid substitution on the structure and function of a human protein using straightforward physical and comparative considerations; http://genetics.bwh.harvard.edu/pph2/).

§ Allele frequency across multiple populations from Genome Aggregation Database (GnomAD: https://gnomad.broadinstitute.org/).

‖ Splicing predictor (Splice prediction by Neural Network; https://www.fruitfly.org/seq_tools/splice.html).

¶ ClinVar (archive of reports of the relationships among human variations and phenotypes, with supporting evidence; https://www.ncbi.nlm.nih.gov/clinvar/intro/) or Uniprot (high-quality and freely accessible resource of protein sequence and functional information; https://www.uniprot.org/).

Pat-, pathogenic; Lik. Pat., likely pathogenic; Unc, unconclusive.

## Results

### Demographic Information

A total of 67 patients with biallelic disease-causing variants in the *BBS1* and *BBS10* genes were included. Thirty-eight of all patients with BBS had presumed pathogenic variants in *BBS1* (52% female patients and 48% male patients) and 29 in *BBS10* (50% female patients and 50% male patients). The majority of the cohort was of Caucasian ancestry with less than 1% of Asian origin (India/Pakistan). The information about consanguinity was not reported in the patient charts. The age of patients at their first visit ranged from 2 to 49 years with mean duration of observation time of 9.7 years and mean number of 6 visits (see Table 1).

### Electrophysiological Phenotype

Overall, data from ERG assessments were available for 51 patients (76%; *n* = 35 for *BBS1*, and *n* = 16 for *BBS10*). Seven patients with *BBS1* (20% of patients with *BBS1*) had a non-recordable ERG (mean age = 22 years), whereas this was in 5 out of 16 (31%) patients with *BBS10* (mean age = 16 years).

**Figure 1.**
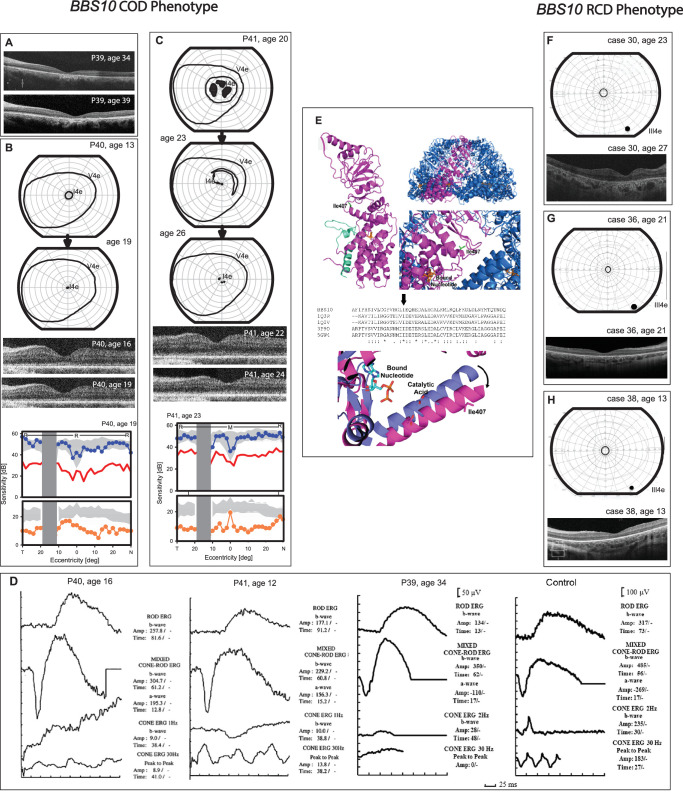
**Phenotypes of** **patients with** ***BBS10*** **with COD compared to *BBS10* with RCD.** (**A**) Optical coherence tomography (OCT) of case 39 at the corresponding ages of 34 and 39 years showing thinning of the retina and atrophy in the central macula with relatively preserved photoreceptors outside of this area. (**B**) Goldmann visual fields results (top) in patients P40 at different ages to I4e and V4e isopters showing preserved fields to the V4e isopter and significant field loss to I4e. (*Center*) OCT images through the fovea at different ages. (*Lower panels*) Normal dark-adapted two-color static perimetry profiles across the horizontal meridian; light-adapted profiles show measurable but reduced cone function across 60 degrees of the profile. (**C**) Same order of phenotyping for P41. (**D**) ERG of the 3 COD cases; three first ERG tracings are of patients: P40 at age 16 years, P41 at age 13 years, and P39 at age 34 years. The fourth (*right*) is that of a subject with normal visual function (control). DA ERGs (two upper traces for each patient ERGs) are within normal limits representing normal rod photoreceptors function in the three patients. LA ERGs (two lower traces for each patient ERGs) are reduced representing severely attenuated cone function in these patients. Taken together, these ERGs are consistent with cone dystrophy. DA, dark adapted; LA, light adapted; ERG, full field electroretinogram; X axis, time in msec; Y axis, amplitude µV. (**E**) Modeling of two of the COD variants were created from the mammalian chaperonin TRiC/CCT (PDB ID 3IYG) (5). Upper panel; (top left) Monomer structure showing the position of Ile407 (*green sticks*) and bound nucleotide (ADP, *orange sticks*). The small translated portion of the monomer that remains with the Glu61* alteration is shown in *light green*, (*top right*) oligomeric ring chaperonin structure with magnification at Ile407. Ile407 is shown to lie at an intersubunit interface near the bound ADP (*orange sticks*), the close-up of Ile407 residue position illustrates proximity to the central catalytic cavity and bound nucleotide. *Middle panel*; ClustalW alignment of selected chaperonin sequences showing conservation of the Ile407 and following acidic residue (Glu in BBS10). *Lower panel*; Comparison of open and closed forms of chaperonins. Movement of the Ile407 helix is illustrated between the open (*purple*) and closed (*magenta*) forms of GroEL/GroES. The catalytic acid of chaperonins is shown as sticks, in proximity to bound ADP analog (present in solved structure). After ATP hydrolysis, the helix rotates and moves as part of the twist mechanism of facilitated substrate protein folding that the chaperonin catalyses. Ile407 is on the opposite end of the helix, and is shown as stick-like structures. (**F–H**) Examples of three patients with *BBS10*-RCD at different ages.

Of the *BBS1*-recordable ERGs, 22 (78%, 7.8–27 years) showed RCD and 6 cases (21%, 15.1–35.2 years) showed a CORD phenotype. Of the 11 (69%) recordable *BBS10*-ERGs, an RCD pattern was documented in 8 cases (73%, 4–16.3 years) whereas 3 patients with *BBS10* (cases 39, 40, 41) had a stable or very slowly progressive COD phenotype (see Table 1). For these 3 patients with *BBS10-*COD, light-adapted (LA) photopic ERGs were severely reduced at the mean age of 22.3 years (12–39 years) and rod responses were normal (Fig. 1D). In Case 39, ERGs performed at ages 34 and 39 years showed no progression.

### Visual Acuity

**Figure 2.**
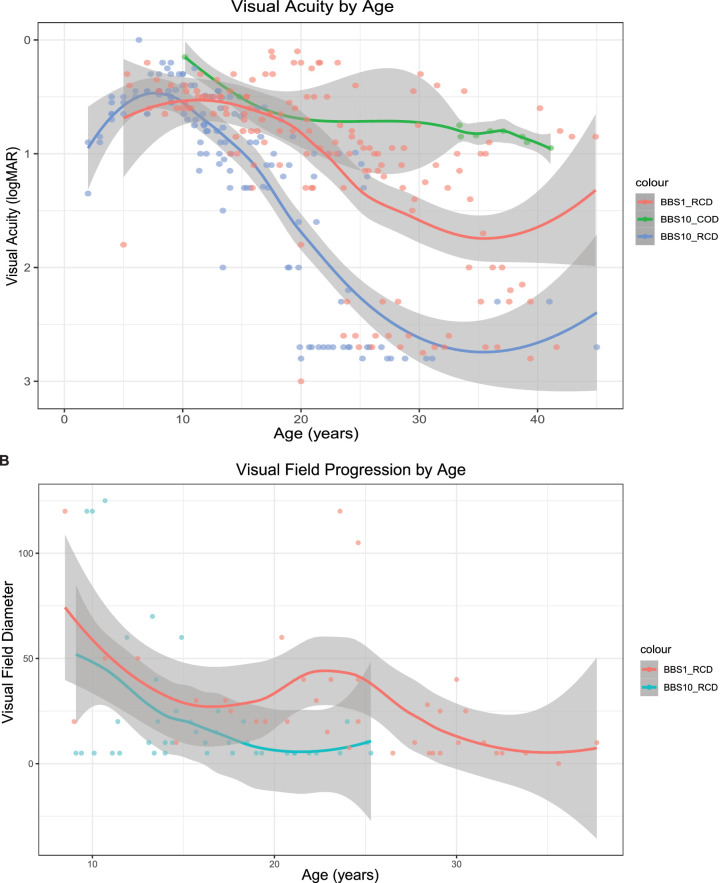
**Difference in the visual acuity and visual field changes in between** patients with ***BBS1*** **and** patients with ***BBS10*** **over time.** (**A**) Comparison among patients with *BBS1*, patients with *BBS10*-RCD, and patients with *BBS10*-COD. Blue trend line corresponds to patients with *BBS10* with RCD, red trend line to patients with *BBS1*, and green trend line to patients with *BBS10* with COD. Each dot represents VA results for each patient in the three BBS cohorts. Visual acuity had a linear decline over time in each cohort with patients with BBS10-RCD showing the fastest change, followed by patients with *BBS1*. The slowest progression was observed in patients with *BBS10* with COD. VA, visual acuity; LogMAR, the Logarithm of the Minimum Angle of Resolution; RCD, rod-cone dystrophy; COD, cone dystrophy. (**B**) Change in GVF diameter to III4e isopter in all patients with *BBS1* and patients with *BBS10-*RCD dystrophy. Patients with *BBS10*-RCD had more constricted visual fields to III4e stimuli earlier compared to patients with *BBS1* of the same age. GVF, Goldmann visual field; RCD, rod-cone dystrophy. Red trend line corresponds to patients with *BBS1* and blue trend line to patients with *BBS10*-RCD. Each dot represents the mean diameter (right and left eyes) of available GVF to III4e isopter.

A total of 139 VA measurements (mean 5/patient, range of 1–15) were available for *BBS1* (age ranged = 5–47.8 years); and 143 VA measurements (mean 5/patient, range of 1–13) for patients with *BBS10* (5–41 years). Not all measurements were available from all subjects at all visits. In the first decade, the VA profiles of patients with *BBS1* and patients with *BBS10* were similar, after which the rate of VA decline increased earlier in the *BBS10* cohort (approximately 15 years) than in the *BBS1* cohort (approximately 25 years; Fig. 2A). The greatest vision loss was documented in the late teenage years (*BBS10*) and early adulthood (*BBS1*).

A very slow progression in VA loss was observed in three patients with *BBS10* with COD. At the mean age of 20 years, VA for patients with *BBS10-*COD and patients with RCD were 0.5 LogMAR and 1.5 LogMAR, respectively; whereas for patients with *BBS1* at the mean age of 20 years, the mean VA was 0.85 LogMAR. One patient with *BBS1* developed NLP vision (2.9 LogMAR) at the age of 16 years, before which time he recalled symptoms of nyctalopia. VA of LP (2.8 LogMAR) was reported in 2 patients with *BBS1* at the mean age of 40.3 years and 4 patients with *BBS10* at the mean age of 25 years (20–27 years).

The patients with *BBS10*-COD had different levels of reduced visual acuity (case 39; 0.95 LogMAR age 41 years, case 40; 0.5 LogMAR age 14.8 years and case 41; 0.15 LogMAR age 10.2 years) suggesting a slow decline over time though patients reported VA to be stable.

### Visual Fields

Goldmann VFs were available for 20 (69%) patients with *BBS1* (9–47.8 years); and 14 (50%) patients with *BBS10* (9–38.6 years). Isopters used were either III4e, V4e, or combined. The sample size of responses to V4e was small, hence not shown.

Although there was variability between the different genes and mutations involved and within age groups, there was a clear inverse relationship between age and VF diameter for both III4e and V4e isopters in patients with *BBS1* and in patients with *BBS10-RCD* (see Fig. 2B). Despite the variability, as observed for the visual acuity changes, visual field narrowing in patients with *BBS10* was earlier and somewhat greater than in patients with *BBS1*.

In patients with *BBS10* with RCD, the III4e isopter became unrecordable by the age of 25 years, whereas at the same age, patients with *BBS1* had on average ≥ 20 degrees of preserved field to this isopter. Although GVFs in patients with *BBS10* with RCD were not detectable to either the III4e or V4e stimulus at the age of 33, 10 of 13 (77%) of patients with BBS1 aged 30 years or older had a recordable field (range of 5–40 degrees to the III4e, IV4e, or V4e stimuli [average 20 degrees]).

At the age of 18 years GVF to V4e was similar for both cohorts, followed by a more rapid decline of GVF to V4e after age of 20 years in patients with *BBS10* with RCD. The oldest patients with *BBS1* with a recordable field was 44.9 years, whereas for patients with *BBS10* it was 25 years.

Examples of the *BBS10*-RCD phenotype are shown in Figures 1F–H contrasting with that of *BBS10*-COD cases (see Figs. 1B, 1C top). Dark-adapted chromatic horizontal static perimetry profiles performed only in patients with *BBS10* 40 and 41 were within normal limits while light-adapted profiles showed measurable but reduced cone function across 60 degrees (see Figs. 1B, 1C lower panels).

### The “Systemic” BBS Phenotype may be Very Subtle

All participants except the three cases of *BBS10*-COD (cases 39–41) had one to several extraocular features reminiscent of BBS. The molecular diagnosis of the three patients with *BBS10*-COD was from retinal degeneration gene panel testing as they were not suspected of having BBS. The availability of information about common systemic BBS features was variable (see Table 2).^18^

The most prevalent extraocular features were digital anomalies (postaxial polydactyly, syndactyly, or brachydactyly), present in 96% of patients with *BBS1* and 82% of patients with *BBS10*; followed by developmental delay, poor coordination, and kidney and liver anomalies. Because data were not available for every feature in each patient, an estimation of the frequency of each sign was impossible. Some details on part of this cohort were previously published.^2^^–^^4^^,^^22^^,^^50^^,^^51^

### Refractive Errors Were Present in 90%

Half of the 21 documented patients with *BBS1* were myopic whereas the other half were hyperopic. Twelve (57%) individuals had significant astigmatism (>2 D) in combination with either myopia or hyperopia. For patients with *BBS10*, myopia was present in 16 (69%) of the 27 documented cases, whereas emmetropia was only seen in 3 (13%) and astigmatism was observed in about half (46%). The myopic skew in patients with *BBS10*-RCD compared to patients with *BBS1* was statistically significant (*P* = 0.046). The three patients with *BBS10*-COD were also myopic (spherical equivalent: −8.0 D, −1.5 D, and −4.5 D, respectively).

### Nyctalopia was Common by the First Decade and Cataracts by the Second Decade

Nyctalopia was an early symptom except in the three *BBS10-COD* cases. Only the eldest patient with *BBS10*-COD experienced photophobia at the age of 36 years.

Cataracts were documented at the mean age of 18.4 years in patients with *BBS10*-RCD (*n* = 18) compared to 27.4 years in patients with *BBS1* (*n* = 24; see Table 1).

### Features of Retinal Degeneration are not Specific to BBS

Retinal features in both *BBS1* and *BBS10*-RCD cohorts showed advanced retinal degeneration with optic disc pallor, blood vessel attenuation, retinal thinning, and maculopathy, as published previously and are not specific to BBS.^2^^,^^26^

OCT showed loss of structural integrity and markedly thinned outer retina (see Figs. 1F–H) and FAF had a characteristic granular pattern as previously-reported.^2^^,^^9^^,^^52^ Whereas the *BBS10*-COD phenotype presenting with a maculopathy, as in case 39, had well-maintained retinal lamination compared to patients with *BBS10*-RCD of similar ages (see Fig. 1).

### Genetic Analyses

Participants had confirmed biallelic variants in *BBS1* or *BBS10* genes, which included 11 novel variants (32%). In the 38 patients with *BBS1*, there was a previously documented enrichment for a common missense variant c.1169T>G, p.Met390Arg present in 31 (81.5%) patients, of whom 24 (63%) were homozygotes (see Table 2).^9^^,^^52^ It is known that 80% of Caucasian patients carry this missense variant.^53^ There are suggestions that this variation is a result of “hot-spot” in the gene and is the effect of multiple mutations having occurred independently at the same nucleotide. Therefore, there is a possibility that c.1169T>G, p.Met390Arg happened once, a long time ago, and was spread by emigration from its source community.^54^^,^^55^

The *BBS10* cohort was characterized by a total of 22 presumed pathogenic variants, showing more allelic heterogeneity than seen in the patients with *BBS1*. The c.271dup, p.(Cys91Leufs*5) and c.145C>T, (p.Arg49Trp) variants were by far the most common variants.

Two brothers with *BBS10*-COD (cases 40 and 41) were compound heterozygotes for novel variants: c.226C>T, p.(Leu76Phe) and c.181G>T, p.(Glu61*); whereas the third patient was homozygous for missense variant c.1120T>C, p.(Ile407Thr), which is rare and was seen once as heterozygous in a patient with BBS, but never observed as homozygous. Previously, the variant c.226C>T, p.(Leu76Phe) was reported as a compound heterozygote and predicted damaging, while c.181G>T, p.(Glu61*) is novel, not reported in ClinVar and gnomAD. As the phenotype was different, protein modeling was performed to further validate pathogenicity of c.1220T>C, p.(Ile407Thr) and c.181G>T, p.(Glu61*) using the mammalian chaperonin TRiC/CCT (PDB 3IYG; see Fig. 1E).^46^ The models produced (Phyre2) had a confidence score of 100, with 22% sequence identity, and 41% sequence homology. This type of chaperonin, an ATPase, forms oligomeric homomultimers (double ring hexadecamers) as functional units. Because the highly conserved p.(Ile407) is present at an intersubunit interface, a mutation to threonine could alter how ATP hydrolysis induces the protein conformation changes, which is vital to chaperone function.^47^ The Glu61* is predicted to cause a null protein monomer, making it impossible for the large multimeric structure to form. A completely nonfunctional protein would result. It is possible that the milder phenotype associated with the Ile407 and Leu76Phe variants related to a milder effect on *BBS10* function that among the other mutations noted which were mostly null.

## Discussion

Although patients with BBS are known to have variable phenotypic severity, retinal degeneration is the feature always present, is relentlessly progressive and leads to legal blindness in late teenage years or young adulthood. Little was known about the natural history in molecularly characterized patients. This large genotyped cohort of patients allowed comparison of the natural history of vision loss related to the most commonly involved genes, *BBS1* and *BBS10*, together accounting for over 40% of BBS cases.^4^^,^^6^^,^^56^

Murine studies have been useful in further understanding BBS phenotypes as the genetic subtypes largely recapitulate the human phenotype.^57^^–^^59^ Recent work by Kretschmer et al.^60^ showed retinal degeneration phenotype differences among *Bbs5*, *6*, and *8* mice, with *Bbs8* deficient mice showing the fastest rate of retinal degeneration. In contrast, the loss of *Bbs5* (another BBSome component) showed very little degeneration. The retinal degeneration in the *Bbs10^−^^/^^−^* mouse model has recently been documented to progress more rapidly and to be more severe than *Bbs1^M390R^* based on functional vision measured by a visually guided swim assay, paralleling what our current study found in humans (A.V. Drack MD, personal communication, October 2020; Drack AV, et al. IOVS 2021; 62: ARVO E-Abstract 1178).

### What did we Learn About Visual Function in Patients With BBS-related Disease?

In the last 25 years, we learned that BBS is a genetically heterogeneous group of disorders with phenotypic and molecular overlap with other ciliopathies. Efforts to characterize the ocular phenotype of clinically defined cohorts of patients with BBS revealed important basic characteristics of BBS, although these may not reflect BBS gene-related disease subtype.^30^^,^^32^^,^^33^^,^^52^^,^^61^^–^^65^ For example, nyctalopia is an early symptom, photophobia is variable and manifests at different ages, and the majority of patients were reported to be myopic and develop cataract in early teenage years. The early phase of the disease can be missed as it is often *sine pigmento*, and the fundus changes do not have BBS-specific characteristics.^32^^,^^64^ Riise^30^^,^^31^ and Fulton et al.^34^ evaluated visual function changes in young cohorts and reported variability in VA decline and severe loss of VF (*n* = 18),^64^ which supports our observations, except that we see differences between patients with *BBS1* and patients with *BBS10*. ERG recordings indicated early involvement of rod photoreceptors which is similar to our findings.^32^^,^^63^^,^^64^

In our cohort, when the ERG was recordable, we report a predominant RCD phenotype (78% *BBS1* and 85% *BBS10*), a CORD phenotype in patients with *BBS1* and a COD phenotype only in patients with *BBS10*. Cone dystrophy was previously reported in a patient with a systemic BBS phenotype and variants in *BBS6*, unlike our COD cases.^29^ Other cases of non-syndromic *BBS*-related RCD disease were previously reported,^27^^,^^66^ but we report for the first time a pure cone dystrophy phenotype in only one case with hand polydactyly.

In our study, refractive errors are common in BBS and correction often benefits the patients despite the retinal degeneration; myopia was most prevalent in patients with *BBS10*. Comparing patients with *BBS1* to patients with *BBS10*, there was a significant difference in changes in visual acuity and visual field, changes being more severe and earlier in patients with *BBS10*.

Our work supports previous studies that suggested that pathogenic variants in chaperonin-like genes (*MKKS/BBS6*, *BBS10*, and *BBS12*) usually lead to a more severe phenotype than those with changes affecting BBSome components, such as *BBS1.*^4^^,^^20^^,^^67^

Strengths of our study include the large, balanced, cohort size of genotyped patients, the report of a novel COD phenotype in 3 patients with *BBS10-*related retinal degeneration, the longitudinal data available and that the data was captured at a wide range of ages. However, availability of the data varied at each site in part owing to the fact that no formal guidelines exist for the evaluation of these patients unlike what was recently developed for Joubert syndrome.^68^ In addition, the cognition and/or behavioral characteristic of some patients would not always allow comprehensive testing. These factors together with the allelic heterogeneity precludes any mutation-phenotypic interpretation.

We believe that a prospective study would better capture uniform health parameters, but these carry time- and cost-related limitations as most inherited retinal diseases progress over years. Our multicenter retrospective approach provided valuable information in a reasonable time frame. With the developments in gene therapy in *Bbs* mouse-models: improving the electrophysiological responses in *Bbs4*-/- mice, and the recent success in rescuing function in the *Bbs10* model by sustainable effect on the improvement of rod- and cone responses over 1 year,^69^^–^^71^ 2002 (Drack AV, et al. IOVS 2021; 62: ARVO E-Abstract 1178) the recent identification of a naturally occurring non-human primate model of BBS (type 7)^72^ and the success of *RPE65* gene replacement therapy,^73^ there is enthusiasm and hope to make *BBS*-related retinal degeneration a treatable condition.

Our retrospective study on the natural history of visual function in the largest cohort of patients with *BBS1* and patients with *BBS10*, showed that the retinal degeneration time course of *BBS10*-RCD is more rapidly progressive than that of *BBS1*-related disease, which should be considered in the planning of treatment trials for these patients.

In summary, we have highlighted differences between the *BBS1* and *BBS10* phenotypes. The loss in visual function for patients with *BBS10* is earlier and somewhat more severe than for patients with *BBS1*. The gene specific phenotypic differences are supported by data of recent murine studies also showing phenotypic differences among genetic subtypes.^60^ The natural history of the *BBS1* and *BBS10*-related retinal degeneration remains somewhat incomplete as in many cases the age at which the ERG became nondetectable could not be captured and was possibly earlier than documented.
